# Altered insular functional activity among electronic cigarettes users with nicotine dependence

**DOI:** 10.1038/s41398-024-03007-6

**Published:** 2024-07-17

**Authors:** Yunkai Sun, An Xie, Yehong Fang, Haobo Chen, Ling Li, Jinsong Tang, Yanhui Liao

**Affiliations:** 1 Department of Psychiatry, Sir Run Run Shaw Hospital, Zhejiang University School of Medicine, Hangzhou, Zhejiang PR China; 2 Department of Radiology, The People’s Hospital of Hunan Province, Changsha, Hunan PR China

**Keywords:** Addiction, Diagnostic markers

## Abstract

Electronic cigarettes (e-cigs) use, especially among youngsters, has been on the rise in recent years. However, little is known about the long-term effects of the use of e-cigs on brain functional activity. We acquired the resting-state functional magnetic resonance imaging (rs-fMRI) data from 93 e-cigs users with nicotine dependence and 103 health controls (HC). The local synchronization was analyzed via the regional homogeneity (ReHo) method at voxel-wise level. The functional connectivity (FC) between the nucleus accumbens (NAcc), the ventral tegmental area (VTA), and the insula was calculated at ROI-wise level. The support vector machining classification model based on rs-fMRI measures was used to identify e-cigs users from HC. Compared with HC, nicotine-dependent e-cigs users showed increased ReHo in the right rolandic operculum and the right insula (*p* < 0.05, FDR corrected). At the ROI-wise level, abnormal FCs between the NAcc, the VTA, and the insula were found in e-cigs users compared to HC (*p* < 0.05, FDR corrected). Correlation analysis found a significant negative correlation between ReHo in the left NAcc and duration of e-cigs use (*r* = −0.273, *p* = 0.008, FDR corrected). The following support vector machine model based on significant results of rs-fMRI successfully differentiates chronic e-cigs users from HC with an accuracy of 73.47%, an AUC of 0.781, a sensitivity of 67.74%, and a specificity of 78.64%. Dysregulated spontaneous activity and FC of addiction-related regions were found in e-cigs users with nicotine dependence, which provides crucial insights into the prevention of its initial use and intervention for quitting e-cigs.

## Introduction

Electronic cigarettes (e-cigs) are novel vaporizing devices that deliver nicotine with battery power. Due to its potential as an alternative to smoking cessation and its appeal to teenagers, the number of e-cigs users is rapidly increasing in many countries [1]. In European countries, about 30% of current smokers ever used e-cigs [2]. In the United States, the rate of e-cigs use among high school students was 19.6%, while it was 4.7% among middle school students [3, 4]. A survey conducted in China revealed that approximately a quarter of young Chinese adults have ever used e-cigs [5]. Chronic use of e-cigs may increase the risk of developing nicotine dependence and have negative effects on psychological well-being such as increased depressive symptoms, decreased sleep quality and so on [6–8]. Given the dramatic increase in e-cigs use in recent years, there is growing concern about the associated adverse effects. However, the impact of repeated use of e-cigs on brain structure and function remains largely unknown. Such information may provide valuable insights for developing programs aimed at optimizing the prevention and treatment of e-cigs dependence.

Some studies support e-cigs as a valid alternative for quitting combustible cigarettes, while others suggest that repeated use of e-cigs may have negative health effects [6, 9]. The effects of e-cigs on lung and microvascular function are similar to or even greater than those of conventional cigarettes [8, 10]. For instance, a study on healthy participants revealed that lung perfusion increased after exposure to e-cigs [11]. Another study showed that healthy nonsmokers exposed to e-cigs experienced increased resistivity index and aortic pulse wave velocity [12]. Additionally, exposure to e-cigs has been also found to exhibit neurotoxic effects on brain [13, 14]. Animal experiments have revealed neurotoxic effects associated with e-cigarette aerosol exposure, including increased expression of α-7 nicotinic acetylcholine receptors (nAChRs) in the prefrontal cortex and striatum, reduced expression of glutamate transporter-1 in the striatum [15], increased oxidative stress in the prefrontal cortex [16], elevated metal levels in the prefrontal cortex and striatum [16], disruption of the blood-brain barrier integrity, and promotion of neurovascular inflammation [17], reduced brain glucose uptake and glucose transporters [18]. Moreover, exposure to e-cigs exhibited potentially harmful neurodevelopmental effects during early pregnancy or early-life exposure. For example, maternal exposure to e-cigs containing nicotine had been shown to increase offspring bodyweight and impair motor skill learning [19].

However, the impact of e-cigs, especially in the long-term effect, on the brain functional activity of individuals with nicotine dependence remains unknown. E-cigs likely promote the same addictive behaviors as traditional combustible cigarettes, resulting in increasing chronic and repeated use [20]. Studies found adolescents who have ever used e-cigs were more likely to use combustible tobacco compared with nonusers [21]. E-cigs users exhibited higher levels of nicotine dependence than traditional tobacco smokers [20, 22]. Existing evidence suggested e-cigs use, especially long-term use, may lead to human brain functional activity alterations.

Resting-state functional magnetic resonance imaging (rs-fMRI) is a useful and noninvasive tool to measure human brain functional activity [23]. So far, studies using fMRI were focused on measuring the acute effects of e-cigs intake in the brain with a small sample. A study with 9 participants found abnormal functional connectivity (FC) between the insula and prefrontal cortex following e-cigs use [24]. Another study found youth who had tried e-cigs showed higher activation in the nucleus accumbens during viewing advertisements about sweet or fruit flavor e-cigs [25].

The current study aimed to measure brain functional activity alternations among e-cigs users who have nicotine dependence/addiction. Studies on substance dependence have begun to suggest more precise links between functional properties of some brain regions (including the insula, the nucleus accumbens (NAcc), and the ventral tegmental area (VTA)) with initiation and maintain of addiction [26, 27]. Some of these findings may be relevant to understanding the potential effects of repeated use of e-cigs. In this study, we measured regional homogeneity (ReHo) map at voxel-wise level, and FC between the NAcc, the VTA, and the insula at ROI-wise level. The relationship between significant functional measures and clinical behavior scales was explored. The support vector machine was used to test if these altered functional activities can classify chronic e-cigs users from health control at the individual level. We hypothesized that (1) chronic e-cigs users showed abnormal brain functional activity in the NAcc, the VTA, and the insula; (2) these abnormal brain regions can be used to classify chronic e-cigs users from HC.

## Methods

### Subjects

We recruited 93 e-cigs users with nicotine dependence and 103 healthy controls via social media advertisement. All subjects were Han Chinese and aged between 18–45. E-cigs users with nicotine dependence need to meet the following criteria: (1) they had used e-cigs containing nicotine for more than one year, and (2) they had a diagnosis of nicotine dependence based on the Diagnostic and Statistical Manual of Mental Disorders (DSM-V) criteria, assessed using the Structured Clinical Interview for DSM Disorders (SCID). Both chronic e-cigs users and healthy control subjects were excluded from the study if they: (1) had learning disabilities or central nervous system dysfunctions; (2) had a history of head injury, psychiatric disorders, or a loss of consciousness lasting more than 10 min; (3) were left-handed or mixed-handed; (4) had undergone electroconvulsive therapy (ECT), brain stimulation therapies or received the anti-psychotic drugs within the last 3 months; (5) had a family history of psychotic disorders; (6) met the criteria for substance dependence (excluding nicotine for subjects in the e-cigs group); (7) were pregnant or had contraindications for MRI.

Subjects underwent cotinine urine dipsticks test to further identify their smoking status with a 200 ng/ml cut-off for urine cotinine. All participants meet the criteria for cotinine levels, with the control group having cotinine levels below the specified cut-off and the e-cigs users having cotinine levels above the specified cut-off. Among all chronic e-cigs users, the study identified 47 individuals who exclusively use e-cigs and 46 individuals who are dual users, using both combustible cigarettes and e-cigs. This classification was based on a criterion of self-reported smoking more than 100 tobacco cigarettes within the last 3 months. All participants completed questionnaires to provide demographic and clinical information, and they also underwent MRI scans.

The study protocol was approved by the Institutional Review Board (IRB) of Sir Run Run Shaw Hospital, Zhejiang University School of Medicine (No. 2022–401–01) and was conducted in accordance with the Helsinki Declaration. All subjects signed and gave written informed consent before the formal experiment.

### Clinical measurements

The demographic characteristics from every subject were acquired, including age, sex, educational level, ethnicity and handedness. Depressive symptoms were estimated by the 21-item Beck Depression Intervention (BDI). For chronic e-cigs users, the characteristics of addictive substance use were also estimated, including tobacco smoking, and e-cigs smoking. Carving for e-cigs was measure by the 10-score Visual Analogue Scale (VAS). Dependence for e-cigs was accessed by the 10-score VAS and the Fagerström Test for Cigarette Dependence (FTCD), respectively.

### MRI data acquisition

All images were acquired using a 3.0 T Siemens Magentom Trio scanner (Allegra; Siemens, Erlangen, Germany) at the Hunan Provincial People’s Hospital, China. The 3D T1-weighted images were obtained through a magnetization-prepared rapid acquisition with gradient echo (MPRAGE) with the following parameters: 176 sagittal slices of 1 mm thickness without gap, TR = 2000 ms, TE = 2.26 ms, FOV = 256 × 256 mm^2^, flip angle = 8°, matrix size = 256 × 256. The rs-fMRI images were obtained using an echo planar imaging (EPI) scan sequence with the following parameters: (TR = 2000 ms, TE = 30 ms, FOV = 220 × 220 mm^2^, flip angle = 90°, 36 axial, 200 volumes). During the acquisition of fMRI data, especially in R-fMRI scans, subjects were asked to relax with closed eyes, think of nothing and not fall asleep.

### Image data processing

All neuroimage data were processed using DPABI (http://www.rfmri.org/), SPM (http://www.fil.ion.ucl.ac.uk/spm/) and written scripts. Firstly, we excluded the first 10 volumes of functional images at the individual level to account for magnetization equilibration effects and participants’ adaptation to the circumstances. Subsequently, the remaining images were temporally corrected for time delay between slices, and aligned to the first volume for head-motion correction. To realign the images, the movement of the participant’s head was calculated by estimating the displacement along each axis and the rotation around each axis for every consecutive volume. Each participant displayed a peak displacement of under 2 mm along all axes and an angular movement of fewer than 2 degrees for each axis. Linear regression method was performed to regress out confounding factors including 24 motion parameters and the average time series of signals from the cerebrospinal fluid and white matter. The images were subsequently normalized to a stereotactic space that conforms to the Montreal Neurological Institute’s standard with a resampling voxel size of 3 mm × 3 mm × 3 mm. Finally, a temporal bandpass filter between 0.01 and 0.08 Hz was applied to the images.

### ReHo calculations

Regional homogeneity (ReHo) is a widely-applied measure to delineate the local synchronization of BOLD signals at the voxel level recorded by rs-fMRI [28]. The ReHo is defined by estimating the relationship between the time series of a given voxel and its spatially adjacent voxels [29]. We calculated the Kendall concordance coefficient of a given voxel with the time courses of 27 neighboring voxels as the ReHo value of a given voxel [28, 30]. For further analysis, the mean ReHo (mReHo) value was estimated by divided by the whole-brain averaged ReHo. The mReHo was spatial smoothed (FWHM = 6 mm). It is noted that the preprocessed steps did not include spatial smooth before the ReHo calculation, to reduce the regional homogeneity among voxels. The ReHo values were extracted specifically from the NAcc, the VTA, and the insular subregions, as described below.

### Functional connectivity analysis

**Fig. 1:**
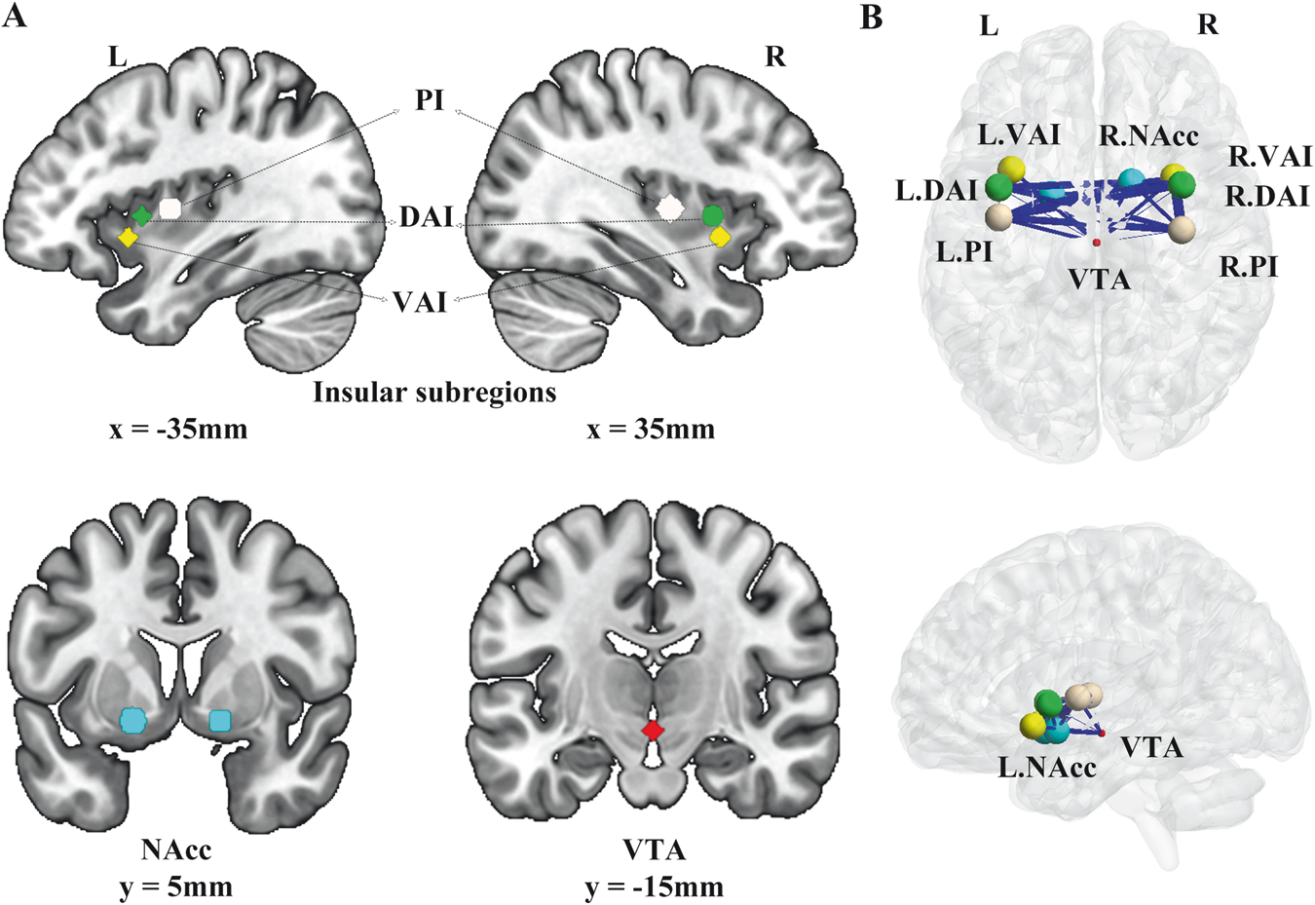
The abnormal functional connectivity in chronic e-cigs users. **A** Eight regions of interest. **B** Aberrant functional connectivity map in chronic e-cigarette users compared to control groups. This map involved the insular subregions (represented by blue circles), the nucleus accumbens (represented by yellow circles), and the ventral tegmental area (represented by red circles). Each abnormal functional connectivity is represented by a blue line, with its thickness indicating the t-value magnitude. L, left; R, right; L.NAcc, left nucleus accumbens; VTA, the ventral tegmental area; L.PI, left ventral anterior insula; L.DAI, left dorsal anterior insula; L.PI, left posterior insula.

We used the Brainnetome atlas to define the bilateral NAcc [31]. For the VTA, we used a spherical seed with a 3-mm radius centered on the ROI at Talairach coordinates (x = 0, y = −16, z = −7) [32]. Due to the functional heterogeneity of the insular cortex [33, 34], we defined six subregions within the insula. These subregions included the following: the left ventral anterior insula (MNI coordinates: x = −33, y = 13, z = −7), the right ventral anterior insula (MNI coordinates: x = 32, y = 10, z = −6), the left dorsal anterior insula (MNI coordinates: x = −38, y = 6, z = 2), the right dorsal anterior insula (MNI coordinates: x = 35, y = 7, z = 3), the left posterior insula (MNI coordinates: x = −38, y = −6, z = 5), and the right posterior insula (MNI coordinates: x = 35, y = −11, z = 6). These subregions were defined by a spherical mask with a 6-mm radius (Fig. 1A).

Functional connectivity (FC) is a method used to examine the functional interactions between different brain areas by measuring the temporal coherence of their activity. In this study, we extracted the mean time series from the identified brain regions. To quantify the FC between these regions, we employed the Pearson correlation method, which measures the linear relationship between two-time series. This resulted in the calculation of 36 functional connections between the selected brain regions. To facilitate further analysis and statistical comparisons, we transformed the correlation coefficients to z values using Fisher’s r-to-z transformation. This transformation helps to normalize the correlation values and allows for more appropriate statistical analyses.

### Statistical analyses

The two-sample t-test and χ^2^ test was used to compare demographic data between the two groups (e-cigs users and HC). To investigate group differences in rs-fMRI measures, we conducted a general linear model (GLM) analysis. Age, sex, and years of education were included as covariates. The rs-fMRI measures included voxel-wise ReHo maps, ReHo of the NAcc, the VTA, and the insular subregions at the ROI level, and functional connectivity between the NAcc and the VTA, as well as the insular subregions. The Pearson correlation method was used to estimate the relationship between the significant findings from ReHo or FC analysis and the characteristic of e-cigs use within chronic e-cigs users. The false discovery rate (FDR) method with a threshold *p* < 0.05 was performed for multiple comparison corrections. To further identify the effect of long-term e-cigs on the brain, we also compare exclusive e-cigs users and dual users in the rs-fMRI measures using GLM with age, sex, and years of education as covariates.

Support vector machine (SVM) is a widely used classifier in neuroimaging research, providing a framework to study brain images at the individual level [35]. In this study, we employed SVM based on rs-fMRI measures that exhibited significant group differences to classify chronic e-cigs users from HC using a leave-one-out cross-validation (LOOCV) approach. To assess the performance of the classifier, the permutation test was performed with 10,000 iterations through randomly relabeling the class labels and recalculating the classification accuracy each time. We considered the classification performance to be reliable if the generalization rate obtained by the classifier trained on the actual class labels exceeded the 95% confidence interval of the classifier trained on randomly relabeled class labels. Furthermore, we calculated the area under the receiver-operating characteristic curve (AUC), sensitivity and specificity to evaluate the performance of the classifier model. Classification measures the area under the receiver operating characteristic (ROC) curve. Sensitivity measures the proportion of true positive samples (e-cigs users) correctly identified by the classifier, while specificity measures the proportion of true negative samples (healthy controls) correctly identified by the classifier.

## Results

### Sample characteristics

Table 1 presents demographics, and clinical characteristics for the total of 93 chronic e-cigs users and 103 healthy controls. We did not find significant group differences between chronic e-cigs users and HC in age and educational level variates. However, there was a significant difference in sex between the two groups (χ^2^ = 19.355, *p* < 0.001). We also found that chronic e-cigs users exhibited higher BDI scores than HC. Age and educational level variates did not differ from exclusive e-cigs users to dual users, while sex showed a significant difference (χ^2^ = 7.355, *p* < 0.001, see Table 1 in supplementary materials).

**Table 1:** Demographic and clinical characteristics of e-cigs users with nicotine dependence and control subjects.

	E-cigs users	HC	T/X	*p*
Number	93	103		
Sex (Male/Female)	67 / 26	42 / 61	19.355	<0.001
Age (M ± SD)	27.47 ± 5.77	28.35 ± 5.33	−1.118	0.265
Edu (years)	15.14 ± 2.63	15.18 ± 3.30	−0.093	0.926
BDI	10.06 ± 8.02	4.70 ± 5.85	5.384	<0.001
E-cigs use variables				
Years of E-cigs (months)	34.76 ± 17.37			
Times for using E-cigs per month	392.63 ± 377.03			
Dependence for E-cigs	6.20 ± 2.32			
Carving for E-cigs	6.28 ± 2.06			
FTCD for E-cigs	3.98 ± 2.82			

Notes: Values are presented as the mean ± SD.

*SD* standard deviation, *E-cigs* electronic cigarettes, *HC* health control, *BDI* Beck Depression Intervention.

### Between-group differences in ReHo

**Fig. 2:**
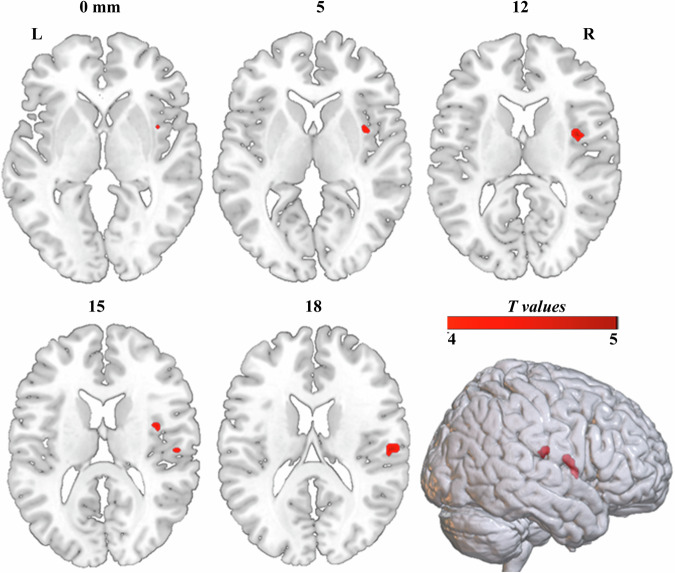
The group difference in voxel-wise ReHo maps between chronic e-cigs users and HC (*p* < 0.05, FDR corrected). L left; R right.

We found that chronic e-cigs users showed significantly increased ReHo in the right rolandic operculum (*p* < 0.05, FDR corrected) and the right insula (*p* < 0.05, FDR corrected) relative to HCs (Fig. 2). There was no compared difference in two significant clusters between exclusive e-cigs users and dual users (*p* > 0.05, FDR corrected).

### Local synchronization of insular subregions, VTA and NAc

**Fig. 3:**
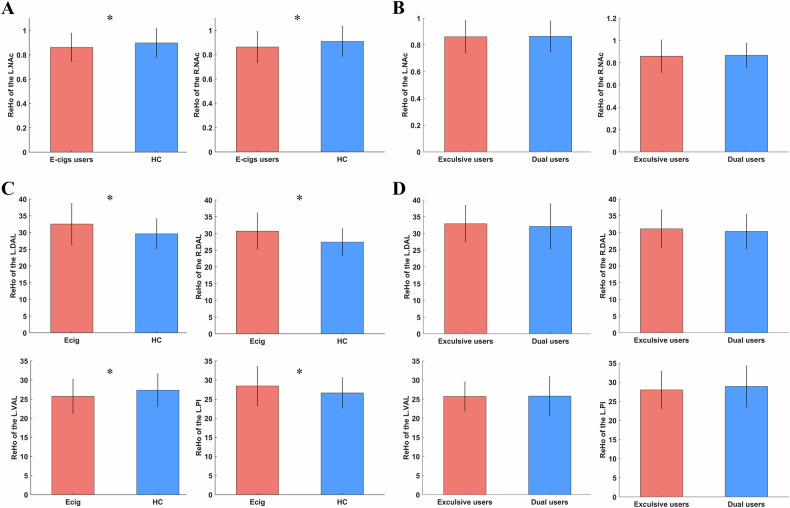
The abnormal local synchronization in chronic e-cigs users. The group difference between chronic e-cigs users and HC **A**, exclusive e-cigs users and dual users in the NAcc **B**. The group difference between chronic e-cigs users and HC **C**, and exclusive e-cigs users and dual users in insular subregions **D**. The significance threshold was *p* < 0.05, FDR corrected. L.NAcc, left nucleus accumbens; R.NAcc, right nucleus accumbens; L.PI, left ventral anterior insula; R.PI, right posterior insula; L.DAI, left dorsal anterior insula; R.DAI, right dorsal anterior insula.

As shown in Fig. 3, we found chronic e-cigs users showed significantly lower ReHo value in the bilateral NAcc (*p* < 0.05, FDR corrected) than HC, while there were no significant changes in the VTA (*p* < 0.05, FDR corrected). With regard to the insular subregions, chronic e-cigs users significantly increased ReHo in the bilateral dorsal anterior insula and the right posterior insula compared to HC, while significantly decreased in the left ventral anterior insula (*p* < 0.05, FDR corrected). However, we did not find any significant group difference between exclusive e-cigs users and dual users (*p* > 0.05).

### Connectivity of the insular subregions, NAcc and VTA

At the ROI-wise level, as described in Fig. 1, the FC between the bilateral NAcc and the VTA in chronic e-cigs users significantly decreased compared to HC (*p* < 0.05, FDR corrected). The functional connectivity of the VTA and all insular subregions showed significant group differences between chronic e-cigs users and HC (*p* < 0.05, FDR corrected). We also found that chronic e-cigs users showed a significantly decreased in the connectivity of the right NAcc with the bilateral dorsal anterior insula, the right ventral anterior insula and the posterior insula when compared to HC (*p* < 0.05, FDR corrected). And significantly decreased functional connectivity between the left NAcc bilateral dorsal anterior insula, the right ventral anterior insula and the posterior insula were found (*p* < 0.05, FDR corrected). Decreased functional connectivity within the subregions of the insular cortex (between the bilateral dorsal anterior insula and the bilateral posterior insula, the right ventral anterior insula and the bilateral posterior insula, the left dorsal anterior insula and the right posterior insula, the left posterior insula and the right posterior insula) was found in e-cigs users compared to HC. However, we did not find any significant group difference between exclusive e-cigs users and dual users (*p* > 0.05).

### The relationship between the brain and e-cigs use characteristics

**Fig. 4:**
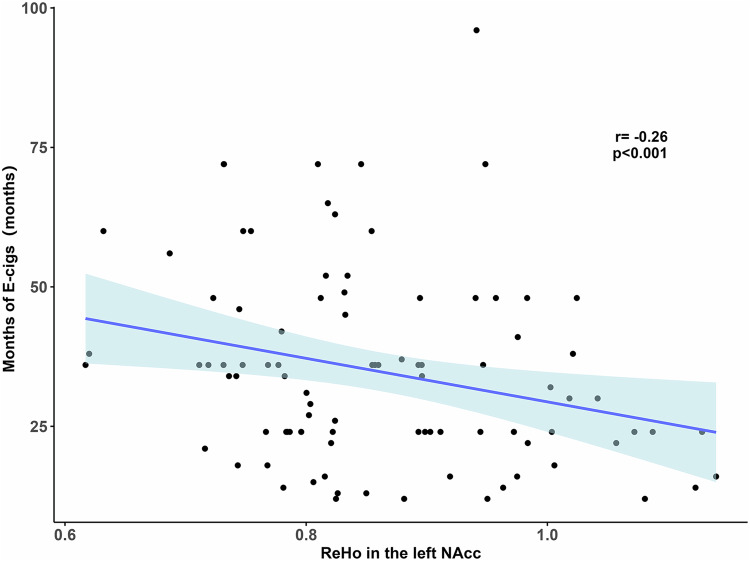
The relationship between ReHo in the left NAcc and years of e-cigs use in chronic e-cigs users.

We found a significant negative correlation between ReHo in the left NAcc and years of e-cigs use within chronic e-cigs users (r = −2.73, *p* = 0.008, FDR corrected, Fig. 4). However, we found no significant associations between rs-fMRI measures and other characteristics of e-cigs use.

### Machine learning analysis

The SVM based on all significant results can differentiate chronic e-cigs users from HC with an accuracy of 73.47%, an AUC of 0.781, a sensitivity of 67.74%, and a specificity of 78.64%. Permutation tests found that the classification accuracy based on actual labels was significantly higher when compared to the random label (*p* < 0.001).

## Discussion

Using a rs-fMRI technique, we identified brain regions with brain functional activity alterations among chronic e-cigs users with nicotine dependence. In the voxel-wise analysis, we observed significant increases in ReHo of the right rolandic operculum and the right insula in e-cigs users with nicotine dependence compared to the HC group. At the ROI-wise level, abnormalities in ReHo were detected in the NAcc and insular subregions. Furthermore, FC analysis revealed significant alterations in connectivity patterns between the NAcc, the VTA, and the insular subregions among nicotine-dependent e-cigs users when compared to HC. The classification model based on these results successfully distinguished chronic e-cigs users from HC. Correlation analysis revealed a negative relationship between ReHo in the left NAcc and years of e-cigs use. Subgroup analysis did not reveal any significant differences between exclusive e-cigs users and dual users. Together, these findings suggested that chronic cigs use alternates brain functional activity.

The finding of altered local synchronization of BOLD signals in the NAcc and the insular subregions associated with chronic e-cigs use was lined with our hypothesis, which showed the impact of e-cigs on brain regions related to addictive behaviors. The NAcc, a key structure within the basal ganglia, plays a role in goal-directed behaviors by integrating information from cortical and limbic regions [36]. Chronic drug exposure disrupts plasticity in the NAcc, leading to an abnormal drive to seek drugs when exposed to drug-associated cues. For example, a positron emission tomography study found altered cue-induced activity in the NAcc among tobacco smokers compared to nonsmokers [37]. The insular cortex, a crucial hub within the salience network involved in processing interoceptive signals and emotions, is associated with nicotine addiction [38]. Altered ReHo in the insula may contribute to the maintenance of e-cigs use. Additionally, we observed altered local synchronized activity in the right rolandic operculum among chronic e-cigs users. These findings align with a VBM meta-analysis demonstrating reduced gray matter volume in the bilateral rolandic operculum, the bilateral prefrontal cortex, and the bilateral insula in addiction-related disorders compared to healthy controls [39].

Altered FC between the insular cortex, the NAcc, and the VTA were found in chronic e-cigs users relative to HC. The insula was able to be segmented into three different subregions with distinct function [40]. The posterior insular cortex was involved in pain perception, sensorimotor processing and language processing [40]. The dorsal anterior insular cortex was responsible for an executive control function, while the ventral anterior insular cortex was associated with social-emotional processing and autonomic function [40, 41]. The unbalance of interoceptive signals originating from physiological states (the posterior insular cortex) and positive hedonic emotions (the anterior insular cortex) was believed to play a key role in the affective learning of the effects of drugs and their associations with specific contexts [42], which was lined with our findings of abnormal internal connectivity within the insula. Abnormal NAcc-VTA connectivity found among e-cigs users are consistent with findings of decreased FC between the VTA and the NAcc in chronic cocaine users [43]. The NAcc and the VTA were crucial components of the mesolimbic reward system, which is widely recognized as playing a key role in the neurobiology of addiction [41]. Dopamine neurons within the VTA projected to the NAcc through the mesolimbic pathway and associations between these regions were essential for drug reward and implicated in incentive motivation, such as substance-related craving [44, 45]. We speculated that reduced functional connectivity between the VTA and the NAcc may represent a vulnerability for the development of e-cigs dependence. The altered connections of the insular subregions and the mesolimbic reward system (the NAcc and VTA) were observed in the current study. Evidence from diffusion tensor imaging (DTI) analysis suggested that the connectivity of the right anterior insula and the NAcc was associated with subsequent relapse to stimulant use [46]. Another study has shown that there is a correlation between the connectivity of the insula and VTA and the severity of nicotine dependence [47]. These findings suggest that the repetitive and sustained use of e-cigs may be linked to the altered FC between the insula, NAcc, and VTA.

We found these altered results were able to successfully differentiate e-cigs users with nicotine dependence from non-users using an SVM classification model. By employing machine learning algorithms, we provided a novel framework to assess disorder-related biomarkers at the individual level [35, 48]. Our findings suggest that the insula, the NAcc, and the VTA may have diagnostic potential for e-cigs dependence. These brain regions appear to play an important role in identifying individuals with a higher susceptibility to develop impulsive drug-seeking behaviors.

Brain and behaviors analysis found ReHo in the left NAcc was negatively related to more long years of e-cigs use. This inverse relationship was consistent with previous studies which revealed brain functional activity in the NAcc was years of addictive substance [43]. Moreover, previous studies have demonstrated that the NAcc plays a crucial role in processing goal-directed actions and mediates the positive reinforcing effects of drugs [49, 50]. Deficiencies in goal-directed behavior have been associated with drug-seeking tendencies and compulsive behaviors, representing notable risk factors for relapse. With prolonged use of electronic cigarettes, it is plausible that this could lead to a decrease in NAcc functional activity, thereby potentially exacerbating drug-seeking behavior [51]. Furthermore, the insular cortex has been recognized as a pivotal predictive factor for anticipating relapse to substance use [46, 52]. We speculated that the left NAcc may potentially serve as a target for neuromodulation, such as through Transcranial Magnetic Stimulation (TMS) [53]. Although some studies using resting-state FC found an inverse relationship between mesolimbic functional activity and subjective craving [32, 54], we did not find similar findings in the current study. One possible explanation is that the subjects in our study had been using e-cigs for over a year, which may have caused a shift from reward-directed behavior to habitual and compulsive behavior [49].

Subgroup analysis found no difference in fMRI indices between exclusive e-cigs users and dual users. Previous studies found that traditional cigarette users, e-cigs users, and dual users all exhibited perceived high stress and depressive mood compared to HC [55, 56]. These results suggest that the impact of exclusive e-cigs use on brain functional activity is comparable to that of traditional cigarettes and e-cigs. Therefore, it is important for smokers, regardless of their specific smoking habits, to quit smoking in order to maximize potential health benefits. Nevertheless, the profiles of brain functional activity observed in this study may serve as markers indicative of an increased propensity for the development of drug use behaviors. These findings contribute to our understanding of the neural mechanisms underlying e-cigs addiction and highlight the potential of utilizing brain imaging techniques for diagnostic purposes.

There are some limitations in this study. First, this study did not include traditional cigarette users. Given that e-cigs users often have a history of combustible cigarette use or engage in dual use [56], future research should compare the effects of e-cigs and traditional cigarettes to better understand their respective impacts on the brain. Second, the current study focused on ROI-wise FC analysis to investigate the effects of chronic e-cigs use on the brain. It would be beneficial to include more brain regions and construct a larger functional network in future studies, as this could provide a more comprehensive understanding of the effects of e-cigs use. Third, while all e-cig users in our study consumed nicotine, it’s important to note that different e-cig brands may contain different substances beyond nicotine. We anticipated that future studies will delve into the effects of these specific substances in e-cigs. Fourth, some studies have reported that white signals may contain functional information, suggesting that signals were not regressed in the processing. Furthers studies should take account into this factor and investigate the temporal properties of white signals. Additionally, while we have employed general linear models to account for the influence of age and sex, we acknowledge that there may still be residual effects of these variables on the outcomes. Given the sample size imbalance (e-cigarette group: 26 females and 67 males), future research should further investigate whether age or sex has an impact on the neural activity associated with e-cigarette use. Finally, while the classification model based on the significant group differences achieved relatively high accuracy, it is important to note that the generalizability of this model is unknown due to the absence of independent test sets. Future studies with larger sample sizes should aim to build a reliable diagnostic model.

In conclusion, this study has identified abnormalities in nicotine-dependent e-cigs users in the functional activity of the NAcc, the VTA, and the insula, which were all linked to the initiation and maintenance of addiction. Although e-cigs can potentially aid smokers in quitting combustible cigarettes, it is important to consider its addiction and changes in brain function associated with their use. Given the insufficiency of existing regulations in China to safeguard youth from e-cigarette usage and nicotine addiction, it is imperative for policy-makers to place a strong emphasis on early prevention of youth e-cigs use.

### Supplementary information

SUPPLEMENTAL MATERIAL

## Data Availability

The data that was used in this study would be available if the readers have reasonable aims to use these data and acquire an agreement from the corresponding author.
