# Supplementary material for: Altered insular functional activity among electronic cigarettes users with nicotine dependence
Source: Transl Psychiatry. 2024 Jul 17;14:293. doi: 10.1038/s41398-024-03007-6 (PMC11255336; doi:10.1038/s41398-024-03007-6)
Supplement: Supplementary file 1 — SUPPLEMENTAL MATERIAL [file 41398_2024_3007_MOESM1_ESM.docx]

**S Table 1 Demographic and clinical characteristics of exclusive e-cigs users and dual users**

|  | **Dual users** | **Exclusive e-cig users** | **T/X** | ***p*** |
| --- | --- | --- | --- | --- |
| **Number** | 46 | 47 |  |  |
| **Sex (Male/Female)** | 39/7 | 28/19 | 7.335 | **0.007** |
| **Age (M ± SD)** | 26.67± 6.00 | 28.36 ± 5.46 | -1.419 | 0.159 |
| **Edu (years)** | 14.73± 2.08 | 15.53 ± 3.01 | -1.473 | 0.144 |
| **BDI** | 11.00 ± 8.74 | 9.14 ± 7.22 | 1.11 | 0.268 |
| **E-cigs use variables** | | | | |
| **Years of e-cigs （months）** | 34.76 ± 17.37 | 34.70 ± 15.82 | 0.0342 | 0.972 |
| **Times for using e-cigs per month** | 349.57 ± 226.00 | 434.79 ± 480.28 | -1.090 | 0.278 |
| **Dependence for e-cigs** | 5.70 ± 2.26 | 6.70 ± 2.30 | -2.127 | **0.036** |
| **Carving for e-cigs** | 5.78 ± 1.92 | 6.77 ± 2.10 | -2.357 | **0.020** |
| **FTCD for e-cigs** | 3.70 ± 2.88 | 4.27 ± 2.76 | -0.992 | 0.323 |

Notes: Values are presented as the mean ± SD.

Abbreviations: SD, standard deviation; e-cigs, electronic cigarettes; HC, health control; BDI, Beck Depression Intervention.
